# Supplementary material for: Longitudinal D-Dimer Trajectories and the Risk of Mortality in Abdominal Trauma Patients: A Group-Based Trajectory Modeling Analysis
Source: J Clin Med. 2023 Jan 30;12(3):1091. doi: 10.3390/jcm12031091 (PMC9917395; doi:10.3390/jcm12031091)
Supplement: Supplementary file 1 [file jcm-12-01091-s001.zip › jcm-2041961-supplementary.pdf]

Supplementary Information

Longitudinal D-Dimer Trajectories and the Risk of Mortality in Abdominal Trauma Patients: A Group-Based Trajectory Modeling Analysis

Chuanrui Sun, BSc[1†], Fengchan Xi, PhD[2,3†], Jiang Li, BSc[1], Wenkui Yu, PhD[3*], Xiling Wang, PhD[1*]

[1] Key Laboratory of Public Health Safety, Ministry of Education, School of Public Health, Fudan University, Xuhui District, Shanghai 200231, China

[2] Research Institute of General Surgery, Affiliated Jinling Hospital, Medical School of Nanjing University, Nanjing 210093, China

[3] Department of Intensive Care Unit, Affiliated Drum Tower Hospital, Medical School of Nanjing Uni-versity, Nanjing 210008, China

[*] Correspondence: yudrnj@163.com (W.Y.); erinwang@fudan.edu.cn (X.W.).

[†] These authors contributed equally to this work.

Table of Contents

S1. Methods **4**

S1.1 Missing data 4

S1.2 Model development 4

S1.3 Individual trajectories and spline curve 5

S1.4 Comparison characteristics between patients with different density of D-dimer 5

S2. Tables **7**

Table S1 Sensitivity analysis of imputation for missing data 7

Table S2 Comparison of model performance for the 4-group models with different shape orders 7

Table S3 Comparison of characteristics in survivors and non-survivors 9

Table S4 Association between trajectory groups and in-hospital all-cause mortality from the sensitivity analysis 11

Table S5 Comparison of baseline characteristics between patients with different density of D-dimer (<3 or ≥3) 11

S3. Figures **13**

Figure S1 Mean D-dimer with 95% CI for distinct trajectory groups with quadratic polynomials from one group to six groups in the first stage of model selection. 13

Figure S2 Nature trajectories of D-dimer in the first 50 days post-trauma. 14

Figure S3 Comparison of distributions for AIS of the abdomen among groups. 15

Figure S4 Receiver operating characteristic (ROC) curves for the D-dimer trajectory and TRISS models in predicting the survival of abdominal trauma patients 18

References **18**

S.1. Methods

S1.1 Missing data

We found that 27 (8.74%) patients had simultaneous missing Abbreviated Injury Scale (AIS) scores of six body regions and Injury Severity Score (ISS). We assumed these data were missing at random as the missing with unknown mechanism was not dependent on observed [1]. Missing AIS scores were imputed by the k-nearest neighbors (KNN) data imputation technique using age, sex, and SOFA score. Missing ISS was calculated by the sum of squares of the highest AIS in each of the three most severely injured body regions for each patient. Sensitivity analysis of imputation for missing data was summarized in Table S1. There were no differences between before and after imputation for AIS scores and ISS.

S1.2 Model development

Group-based trajectory modeling was employed to characterize the longitudinal D-dimer trajectories post-trauma. We used a two-stage model selection process to determine the best-fit number and shape order of trajectories. In the first stage of model selection, we examined models from one group to six groups with quadratic polynomials. Temporal patterns and the Bayesian information criterion (BIC) of each model were presented in Figure S1. Models with more than four groups did not add any distinct clinical features but scaled up model complexity, and the changes in BIC were negligible compared to the model with four groups. Hence, the best-fitting number of groups was four. In the second stage, each group was iteratively fitted with

quadratic, cubic, and quartic polynomials. The best-fit model was chosen based on minimum BIC accompanied by higher entropy (no less than 0.9), proportions of individuals classified in each group (no less than 5%), and the average posterior probability of assignment (no less than 0.7).

S1.3 Individual trajectories and spline curve

A total of 309 patients with 3242 D-dimer measurements within a 50-day time frame post-trauma were included in this study. We plotted the individual trajectories of D-dimer to perform the dynamic changes for each patient. We used natural spline regression to model the average longitudinal changes of D-dimer for all patients (Figure S2). The natural spline curve showed that the mean trajectory of D-dimer levels for all patients demonstrated a high measurement (>5mg/l) on day 0, a marked decline in the first two days post-trauma, a slight increase from day 3 to day 6, and a gradual decline from day 7 to day 50.

S1.4 Comparison characteristics between patients with different density of D-dimer

We compared the baseline characteristics in patients with D-dimer measured at three or over days and patients with D-dimer measured at fewer than three days. We compared age, gender, body mass index (BMI), the Sequential Organ Failure Assessment (SOFA) score, AIS of the abdomen, and ISS between groups. There were missing data for AIS of the abdomen (40.09%) and ISS(40.09%) in patients with D-

dimer measured at fewer than three days, and we did not impute them (Table S5). Patients with D-dimer measured at three or over days had statistically higher SOFA and higher ISS than patients with D-dimer measured at fewer than three days (P <0.001).

S2. Tables

Table S1 Sensitivity analysis of imputation for missing data

Variables	Before imputation	After imputation	P
AIS of the head, median (IQR)	0.00 (0.00,1.00)	0.00 (0.00,1.00)	0.984
AIS of the chest, median (IQR)	2.00 (0.00,3.00)	2.00 (0.00,3.00)	0.805
AIS of the abdomen, median (IQR)	3.00 (2.00,4.00)	3.00 (3.00,4.00)	0.786
AIS of the extremities, median (IQR)	0.00 (0.00,2.00)	0.00 (0.00,2.00)	0.988
AIS of the face, median (IQR)	0.00 (0.00,0.00)	0.00 (0.00,0.00)	0.748
AIS of the external, median (IQR)	0.00 (0.00,1.00)	0.00 (0.00,1.00)	0.991
ISS, median (IQR)	21.00 (16.00,29.00)	20.00 (16.00,29.00)	0.992

Variables were compared using Kruskal-Wallis test between groups. Definition of abbreviation: AIS: Abbreviated Injury Scale; IQR: interquartile range; ISS: Injury Severity Score.

Table S2 Comparison of model performance for the 4-group models with different shape orders

No. of groups	Shape per Group	BIC	Entropy	APPA				Sample Proportion per Group (%)			
				Group 1	Group 2	Group 3	Group 4	Group 1	Group 2	Group 3	Group 4
4	2 2 4 4	-9449.09	0.93	0.98	0.91	0.98	1.00	57.61	28.16	8.41	5.83
	3 2 4 4	-9451.27	0.93	0.97	0.92	0.98	1.00	59.22	26.54	8.41	5.83
	2 3 4 4	-9451.90	0.93	0.97	0.91	0.98	1.00	57.61	28.16	8.41	5.83
	2 4 4 4	-9454.02	0.93	0.97	0.93	0.99	1.00	59.87	25.89	8.41	5.83
	4 2 4 4	-9454.14	0.93	0.97	0.93	0.98	1.00	59.55	26.21	8.41	5.83
	2 2 4 3	-9454.70	0.93	0.91	0.98	0.95	1.00	28.16	57.61	8.41	5.83
	4 3 4 4	-9455.32	0.93	0.94	0.97	0.99	1.00	25.57	60.19	8.41	5.83
	3 2 4 3	-9456.85	0.93	0.96	0.93	0.95	1.00	59.87	25.89	8.41	5.83
	4 3 2 3	-9456.85	0.93	0.95	0.96	0.93	1.00	8.41	59.87	25.89	5.83
	2 3 4 3	-9457.50	0.93	0.98	0.91	0.95	1.00	57.61	28.16	8.41	5.83
	3 3 4 3	-9459.56	0.93	0.93	0.97	0.95	1.00	25.89	59.87	8.41	5.83
	2 4 4 3	-9459.59	0.93	0.97	0.94	0.95	1.00	60.19	25.57	8.41	5.83
	4 2 4 3	-9459.72	0.93	0.97	0.93	0.95	1.00	59.87	25.89	8.41	5.83
	4 3 4 3	-9460.90	0.93	0.94	0.97	0.95	1.00	25.57	60.19	8.41	5.83
	4 4 4 3	-9463.66	0.93	0.97	0.94	0.95	1.00	60.19	25.57	8.41	5.83
	2 3 4 2	-9464.07	0.93	0.98	0.91	0.96	1.00	57.61	28.16	8.41	5.83
	3 3 4 2	-9466.08	0.93	0.97	0.93	0.97	0.97	59.87	25.89	8.09	6.15
	2 4 4 2	-9466.22	0.93	0.93	0.97	0.97	0.97	25.89	59.87	8.09	6.15
	4 4 2 2	-9466.22	0.93	0.97	0.97	0.93	0.97	8.09	59.87	25.89	6.15
	3 4 4 2	-9467.40	0.93	0.97	0.95	0.98	0.97	60.52	25.24	8.09	6.15

4 3 4 2	-9467.40	0.93	0.95	0.97	0.98	0.97	25.24	60.52	8.09	6.15
2 2 3 4	-9509.10	0.90	0.91	0.96	0.94	1.00	25.89	55.66	13.27	5.18
2 3 3 4	-9510.67	0.91	0.97	0.91	0.94	1.00	55.99	25.89	12.94	5.18
2 2 3 3	-9511.57	0.90	0.96	0.91	0.93	1.00	55.66	25.89	13.27	5.18
2 4 3 4	-9512.50	0.91	0.97	0.91	0.94	1.00	56.31	25.89	12.62	5.18
3 3 3 4	-9513.01	0.91	0.91	0.97	0.94	1.00	26.21	55.66	12.94	5.18
2 3 3 3	-9513.22	0.91	0.97	0.91	0.93	1.00	55.99	25.89	12.94	5.18
3 2 3 3	-9513.95	0.90	0.96	0.91	0.93	1.00	55.66	25.89	13.27	5.18
4 2 3 3	-9515.11	0.91	0.91	0.97	0.93	1.00	25.89	56.31	12.62	5.18
4 3 3 4	-9515.88	0.91	0.97	0.91	0.94	1.00	55.66	26.21	12.94	5.18
2 4 3 3	-9516.81	0.90	0.91	0.96	0.93	1.00	25.89	55.66	13.27	5.18
4 4 3 4	-9517.73	0.91	0.91	0.97	0.94	1.00	25.89	56.31	12.62	5.18
3 4 3 3	-9518.43	0.90	0.90	0.97	0.93	1.00	26.21	55.66	12.94	5.18
2 2 3 2	-9520.36	0.90	0.96	0.90	0.94	0.96	55.66	25.89	12.62	5.83
3 3 3 2	-9524.59	0.90	0.97	0.91	0.91	1.00	55.66	25.89	13.27	5.18
3 4 3 2	-9527.46	0.90	0.91	0.97	0.91	1.00	25.89	55.66	13.27	5.18
4 3 3 2	-9527.46	0.90	0.97	0.91	0.91	1.00	55.66	25.89	13.27	5.18
2 4 2 4	-9538.91	0.91	0.97	0.91	0.92	0.95	57.61	26.54	11.00	4.85
3 4 2 4	-9541.19	0.91	0.97	0.91	0.93	0.95	57.93	26.54	10.68	4.85
4 4 2 4	-9543.98	0.91	0.97	0.91	0.93	0.95	57.93	26.54	10.68	4.85
2 3 2 4	-9544.56	0.91	0.97	0.92	0.93	0.96	56.31	27.18	11.65	4.85
3 3 2 4	-9546.67	0.91	0.97	0.90	0.94	0.96	56.63	27.18	11.33	4.85
2 2 2 4	-9550.32	0.91	0.96	0.92	0.95	0.97	56.31	26.86	11.97	4.85
2 2 4 2	-9551.66	0.89	0.87	0.96	0.92	0.95	12.30	60.84	19.42	7.44
2 2 2 3	-9553.04	0.90	0.96	0.92	0.95	0.96	56.31	26.86	11.65	5.18
3 2 4 2	-9554.16	0.89	0.88	0.96	0.90	0.98	11.97	60.84	20.06	7.12
3 3 2 2	-9554.29	0.91	0.97	0.92	0.93	0.96	57.93	25.89	11.65	4.53
3 2 2 3	-9555.40	0.90	0.97	0.91	0.95	0.96	55.66	27.51	11.65	5.18
4 2 2 4	-9555.56	0.91	0.97	0.91	0.95	0.97	55.66	27.51	11.97	4.85
2 3 3 2	-9557.95	0.91	0.90	0.97	0.96	0.97	23.95	60.19	8.41	7.44
2 4 2 3	-9558.27	0.90	0.91	0.97	0.95	0.96	27.51	55.66	11.65	5.18
4 4 4 2	-9560.96	0.90	0.89	0.97	0.92	0.93	11.65	65.37	15.53	7.44
4 2 4 2	-9561.30	0.93	0.92	0.97	0.94	0.94	3.56	62.78	26.21	7.44
2 4 3 2	-9563.49	0.88	0.88	0.96	0.87	0.98	19.09	59.22	14.56	7.12
3 2 3 4	-9563.97	0.88	0.84	0.95	0.92	0.96	12.94	59.55	20.06	7.44
3 4 3 4	-9568.65	0.88	0.85	0.95	0.92	0.96	12.94	59.55	20.06	7.44
3 2 2 4	-9578.16	0.87	0.83	0.95	0.92	0.97	11.65	59.22	21.36	7.77
2 2 2 2	-9579.51	0.87	0.84	0.95	0.91	0.99	11.00	58.90	22.33	7.77
3 2 2 2	-9581.12	0.87	0.82	0.95	0.91	0.99	11.65	59.22	21.36	7.77
2 3 2 2	-9581.86	0.87	0.83	0.95	0.91	0.99	11.33	58.90	22.01	7.77
2 3 2 3	-9582.16	0.87	0.83	0.95	0.90	0.99	11.33	58.90	22.01	7.77
2 4 2 2	-9584.66	0.87	0.83	0.95	0.91	0.99	11.33	58.90	22.01	7.77

3 4 2 2	-9586.28	0.87	0.81	0.95	0.92	0.99	11.97	59.55	20.71	7.77
3 2 3 2	-9591.14	0.94	0.93	0.97	0.93	1.00	3.56	62.46	25.89	8.09
3 3 2 3	-9593.19	0.93	0.94	0.97	0.94	0.95	2.27	60.84	27.51	9.39
4 3 2 4	-9622.34	0.93	0.89	0.98	0.93	0.97	3.24	61.17	27.83	7.77
3 3 4 4	-9623.77	0.95		0.98	0.94	0.98	0.00	70.87	22.65	6.47
3 4 4 4	-9625.96	0.95		0.98	0.94	0.98	0.00	71.20	22.33	6.47
3 4 4 3	-9628.70	0.95		0.98	0.94	0.98	0.00	62.78	28.80	8.41
4 4 4 4	-9630.05	0.95		0.98	0.94	0.97	0.00	62.78	28.80	8.41
3 3 3 3	-9635.67	0.96		0.98	0.94	0.99	0.00	62.14	29.45	8.41
4 2 3 4	-9635.92	0.95		0.98	0.95	0.99	0.00	62.46	29.13	8.41
4 2 3 2	-9637.64	0.95		0.98	0.95	0.99	0.00	62.46	29.13	8.41
4 3 3 3	-9638.54	0.95		0.98	0.94	0.99	0.00	62.14	29.45	8.41
4 4 3 3	-9641.40	0.95		0.98	0.94	0.99	0.00	62.14	29.45	8.41
4 4 3 2	-9642.09	0.95		0.97	0.95	0.99	0.00	62.46	29.13	8.41
4 2 2 3	-9653.71	0.96		0.97	0.95	0.98	0.00	62.14	29.45	8.41
4 2 2 2	-9654.35			0.97	0.95	0.98	0.00	62.14	29.45	8.41
3 4 2 3	-9655.45	0.95		0.98	0.94	0.98	0.00	61.49	30.10	8.41
4 3 2 2	-9656.09	0.95		0.98	0.94	0.98	0.00	61.49	30.10	8.41
4 4 2 3	-9658.32	0.95		0.98	0.94	0.98	0.00	61.49	30.10	8.41

Definition: 2: quadratic; 3: cubic; 4: quartic; BIC: the Bayesian information criterion; APPA: the average posterior probability of assignment.

Table S3 Comparison of characteristics in survivors and non-survivors

Variables	Survivors (n = 286)	Non-survivors (n = 23)	P
**Baseline Characteristics**			
Age, yr, median (IQR)	43.00 (31.00,53.75)	47.00 (38.00,56.50)	0.211
Male gender, n (%)	231 (80.77)	19 (82.61)	>0.999
BMI, median (IQR)	21.97 (20.62,24.02)	23.39 (21.46,27.00)	0.065
**Extra-abdominal trauma**			
Head, n (%)	80 (27.97)	6 (26.09)	>0.999
Face, n (%)	9 (3.15)	0 (0.00)	0.827
Chest, n (%)	175 (61.19)	19 (82.61)	0.069
Extremities, n (%)	92 (32.17)	16 (69.57)	0.001
External, n (%)	77 (26.92)	9 (39.13)	0.31
**Severity of Trauma**			
**AIS**			
Head, median (IQR)	0.00 (0.00, 1.00)	0.00 (0.00, 0.50)	0.733
Face, median (IQR)	0.00 (0.00, 0.00)	0.00 (0.00, 0.00)	0.389
Chest, median (IQR)	2.00 (0.00, 3.00)	3.00 (2.00, 3.00)	0.073
Extremities, median (IQR)	0.00 (0.00, 2.00)	2.00 (0.00, 3.00)	<0.001

External, median (IQR)	0.00 (0.00, 1.00)	0.00 (0.00, 1.00)	0.199
Abdomen, median (IQR)	3.00 (2.00, 4.00)	4.00 (4.00, 5.00)	<0.001
AIS of abdomen >3, n (%)	131 (45.80)	22 (95.65)	<0.001
ISS, median (IQR)	19.50 (16.00,28.50)	34.00 (29.00,39.50)	<0.001
SOFA, median (IQR)	3.00 (2.00,5.75)	14.00 (9.50,16.50)	<0.001
TRISS Ps, median (IQR)	0.98 (0.94, 0.99)	0.82 (0.65, 0.92)	<0.001
**Clinical Treatments**			
UFH, n (%)	258 (90.21)	23 (100.00)	0.232
LMWH, n (%)	94 (32.87)	7 (30.43)	0.993
Blood transfusion, n (%)	150 (52.45)	23 (100.00)	<0.001
Plasma, n (%)	141 (49.30)	23 (100.00)	<0.001
Cryoprecipitate, n (%)	38 (13.29)	17 (73.91)	<0.001
Surgery, n (%)	181 (63.29)	19 (82.61)	0.101
**Hospital Complications and Outcomes**			
VTE, n (%)	23 (8.04)	2 (8.70)	>0.999
Sepsis, n (%)	51 (17.83)	20 (86.96)	<0.001
Intra-abdominal infection, n (%)	37 (12.94)	14 (60.87)	<0.001
Renal dysfunction, n (%)	64 (22.38)	19 (82.61)	<0.001
Liver dysfunction, n (%)	196 (68.53)	22 (95.65)	0.012
LOS, days, median (IQR)	18.35 (11.57,31.50)	18.10 (12.32,25.22)	0.661
**D-dimer Characteristics**			
Maximum D-dimer, mg/L, median (IQR)	7.19 (2.02,16.76)	16.02 (8.21,25.90)	0.004
Mean D-dimer, mg/L, median (IQR)	3.65 (1.17,7.79)	5.91 (3.01,9.58)	0.045
Medium D-dimer, mg/L, median (IQR)	3.11 (1.01,6.48)	5.12 (2.04,7.73)	0.104
Minimum D-dimer, mg/L, median (IQR)	1.42 (0.47,2.63)	1.39 (0.46,2.39)	0.802
SD of D-dimer, mg/L, median (IQR)	1.87 (0.46,4.60)	5.08 (2.30,7.04)	0.004

Definition of abbreviation: IQR: interquartile range; BMI: Body Mass Index; AIS: Abbreviated Injury Scale; ISS: Injury Severity Score; SOFA: Sequential Organ Failure Assessment; TRISS Ps: the probability of survival obtained by the Trauma Injury Severity Score; UFH: unfractionated heparin; LMWH: low-molecular-weight heparin; VTE: venous thromboembolism; LOS: length of hospital stay; SD: standard deviation.

Table S4 Association between trajectory groups and in-hospital all-cause mortality from the sensitivity analysis

Trajectories	Multivariate Analysis	
	OR (95%CI)	P
Group 1	Reference	
Group 2	2.04 (0.51, 8.23)	0.315
Group 3	1.60 (0.27, 9.37)	0.605
Group 4	4.52 (0.83, 24.68)	0.082

Adjusted by BMI, SOFA, and ISS. Group 1: stable low group; Group 2: “moderate-decline”

group; Group 3: “high-rapid decline” group; Group 4: “high-gradual decline” group. OR: odds ratio; CI: confidence interval.

Table S5 Comparison of baseline characteristics between patients with different density of D-dimer (<3 or ≥3)

	D-dimer <3 measurements n =227	D-dimer ≥3 measurements n =309	P
Age, yr, median (IQR)	45.00 (34.00, 54.00)	44.00 (31.00, 54.00)	0.545
Male gender, n (%)	174 (76.65)	250 (80.91)	0.276
BMI, median (IQR)	21.97 (20.76, 23.29)	22.04 (20.66, 24.11)	0.160
SOFA, median (IQR)	2.00 (1.00, 4.00)	4.00 (2.00, 7.00)	<0.001
AIS of abdomen, median (IQR)	3.00 (2.00, 4.00)	3.00 (3.00, 4.00)	0.283
ISS, median (IQR)	16.00 (11.00, 25.00)	20.00 (16.00, 29.00)	<0.001

Only 136 (59.91%) patients had AIS of abdomen and ISS in the group with D-dimer <3 measurements. <3: patients with D-dimer measured at fewer than three days. ≥3: patients with D-dimer measured at three or over days. Definition of abbreviation: IQR: interquartile range; BMI: Body Mass Index; SOFA: Sequential Organ Failure Assessment. AIS: Abbreviated Injury Scale; ISS: Injury Severity Score;

S3. Figures

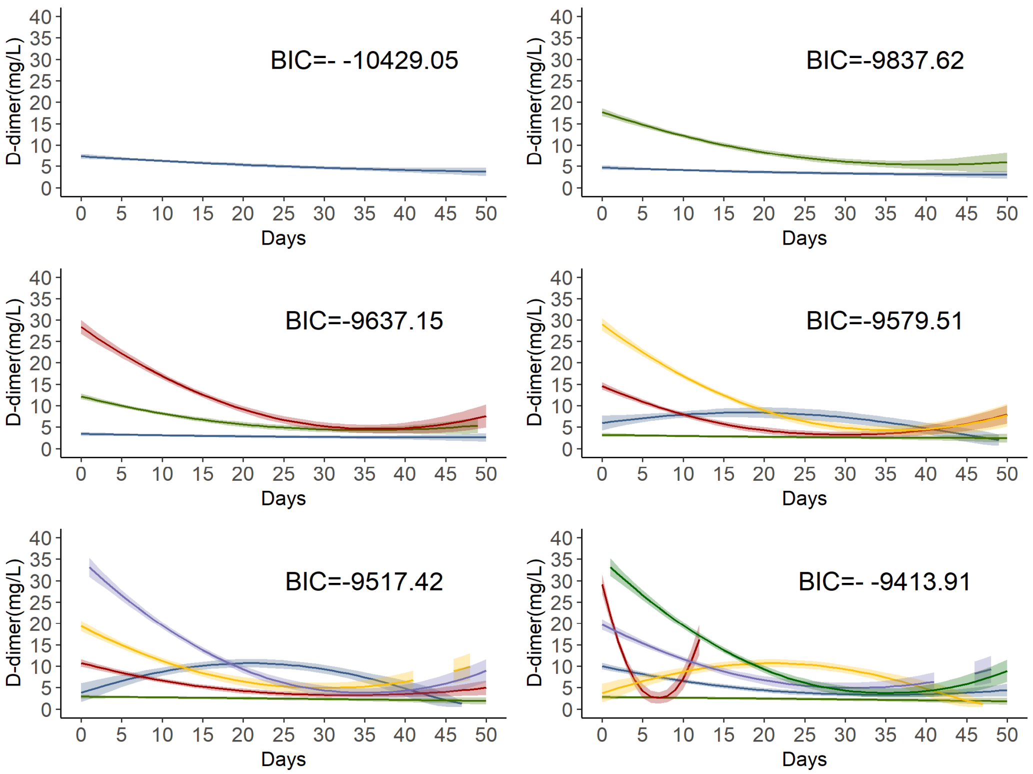

Figure S1. Mean D-dimer with 95% CI for distinct trajectory groups with quadratic polynomials from one group to six groups in the first stage of model selection. BIC: the Bayesian information criterion.

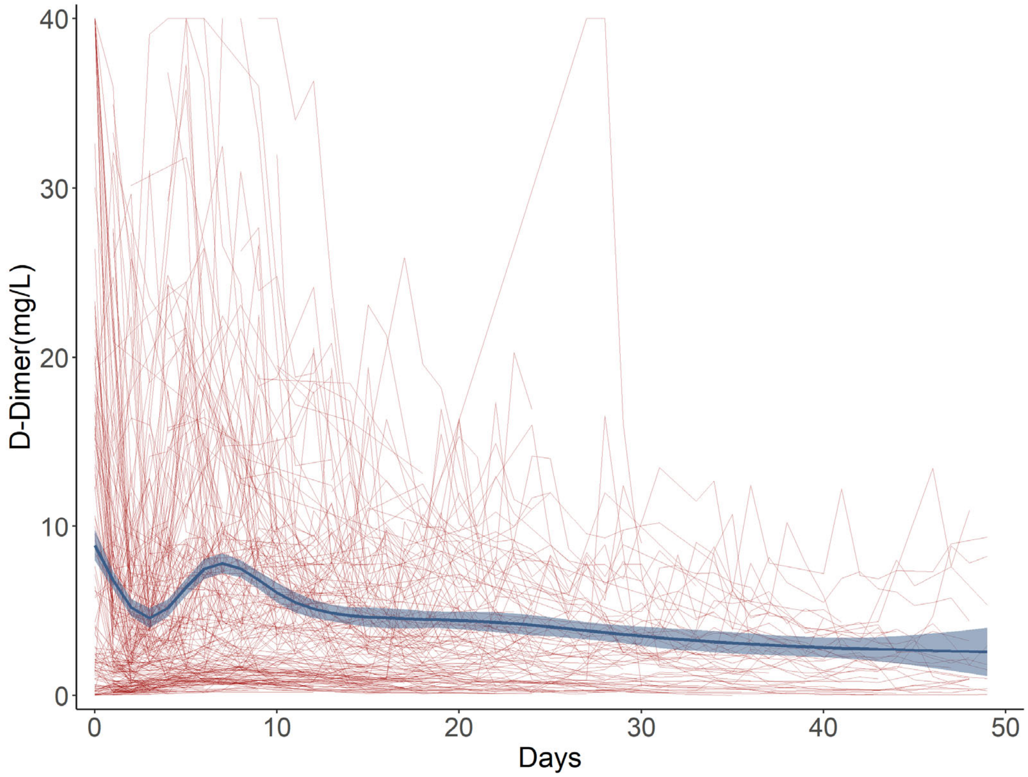

Figure S2. Nature trajectories of D-dimer in the first 50 days post-trauma. Individual trajectories for each patient (red line). Mean D-dimer measurements with the 95% CI on each day (blue line and blue shadow) for all patients, fitted by natural spline regression.

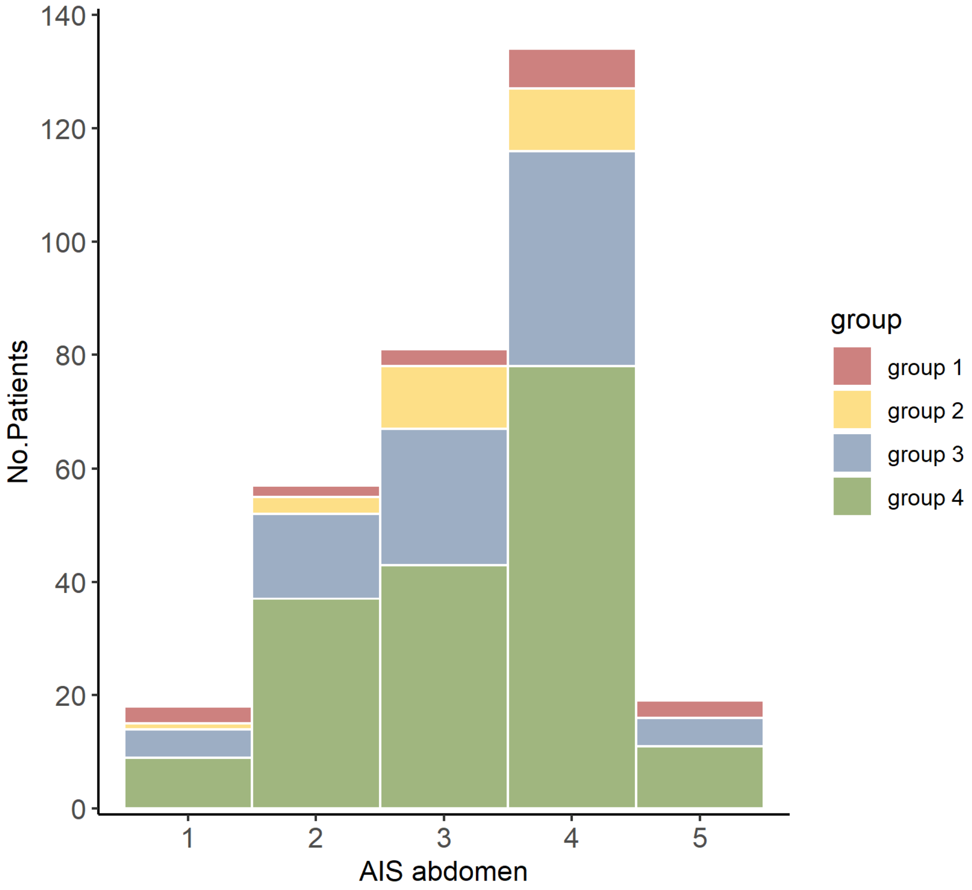

Figure S3. Comparison of distributions for AIS of the abdomen among groups. Group 1: stable low group; Group 2: “moderate-decline” group; Group 3: “high-rapid decline” group; Group 4: “high-gradual decline” group. AIS: Abbreviated Injury Scale.

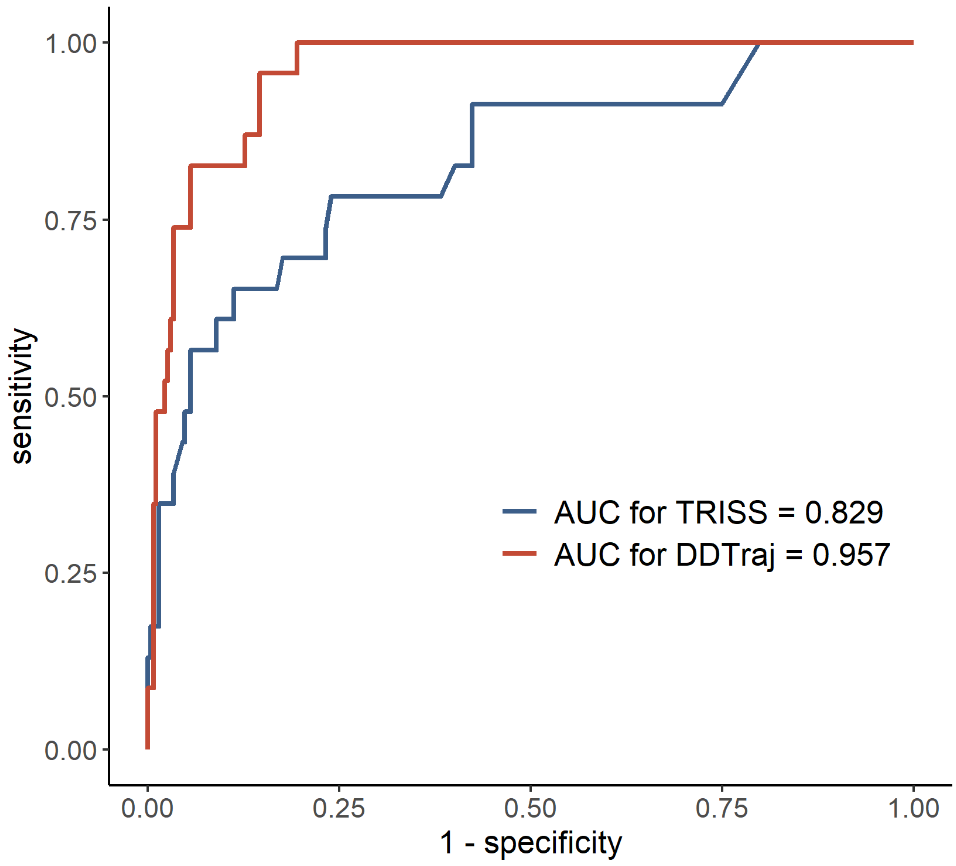

Figure S4. Receiver operating characteristic (ROC) curves for the D-dimer trajectory and TRISS models in predicting the survival of abdominal trauma patients. D-dimer trajectory model was adjusted with BMI, SOFA, and AIS of abdomen. AUC: the area under the receiver-operating-characteristics curve; TRISS: Trauma Injury Severity Score; DDTraj: D-dimer trajectory model.

References

1. Little R.J.A.; Rubin D.B. *Statistical Analysis with Missing Data*, 2nd ed. John Wiley & Sons, Inc.: Hoboken, New Jersey, United States, 2002.
